# Supplementary material for: Physical activity levels in adults and elderly from triaxial and uniaxial accelerometry. The Tromsø Study
Source: PLoS One. 2019 Dec 3;14(12):e0225670. doi: 10.1371/journal.pone.0225670 (PMC6890242; doi:10.1371/journal.pone.0225670)
Supplement: Attachment — Submitted filename: Response to reviewers.docx [file pone.0225670.s001.docx]

**Response to the editor and reviewers**

Additional Editor Comments (if provided):
Congratulations for the authors for this new version of the paper. Based on the assessment of the reviewers and myself, I believe that the paper deserves be published in Plos One. However, some minor points need attention. According to reviewer 2, the paper will benefit from a more detailed discussion on the impact of these kind of measure on public health. Authors should consider expand the discussion on this specific point. Other comment is regarding the title. I will suggest that the authors use terms like "levels" instead prevalence. Why? The paper has 6 tables showing details of the parameters obtained with accelerometer, and only one figure for prevalence. So the component of "levels" in the manuscript is crucial for understanding it.

**Answer:** Thank you for the opportunity for this second revisions to further improve our paper. According to your suggestions, we have further expanded our discussion on the interpretation of our prevalence estimates and how this impacts public health (please see L398-440). We agree on the title change and we have accordingly changed our title to *“Physical activity levels in adults and elderly from triaxial and uniaxial accelerometry. The Tromsø Study”*. We have also included “levels” as a part of our aim: Abstract, see L33 and main text: see L92.

6. Review Comments to the Author

Reviewer #1: I understand your argument when use the reference “Ekelund et al., 2019, BMJ” to justify the use of 150 minutes. In other hand, accelerometry measure includes many decisions not included in self-report. When asked about your habitual physical activity, the answer probably will not include an exact definition of bout or intensity. Thus, if an individual practice sports for 1h, then this 1h will not necessarily be classified as MVPA time in accelerometry (including bout criteria, this will be very different). Therefore, to reach recommendations with accelerometry definitions is very difficult resulting in lower percentages. In addition, prevalence information, in many cases is used to show a health scenario in the world, or in the local context. If you want to keep this kind of measure, I suggest improving the discussion on the differences between prevalence measure by method and how this is used in public health. This measure may be mistakenly used as “the true” about health/physical activity scenario of population and severity of this scenario may vary greatly according to accelerometry decisions. The message of the article is unclear about what to do with this prevalence and how it may impact or apply to public health. Often, “more physical activity” is the final message, but in your case, you show a percentage of population reaching an expected value, so what to do with this percentage so different from previous self-report studies needs further explanations.

**Answer:** Thank you for the suggestion. We agree on the importance of this and we have accordingly further expanded our discussion on the interpretation of our prevalence estimates and how this impacts public health (please see L398-440).

Reviewer #2: Thank you for the opportunity to review this manuscript. I do not have further considerations.

**Answer:** Thank you for reviewing our paper.
